# The Influence of Ethanolic Extract of Hypericum Perforatum (St. John's wort) on Growth Performance, Serum Metabolites, Fat Deposition, Immunity, and Lipid Peroxidation in Broiler Chickens

**DOI:** 10.1002/vms3.70507

**Published:** 2025-07-23

**Authors:** Mohammad Ali Behroozilak, Seyed Adel Moftakharzadeh, Morteza Behroozlak, Vahid Vahedi, Somayeh Bakhshalizadeh

**Affiliations:** ^1^ Animal Science Research Institute of Iran Agricultural Research, Education and Extension Organization (AREEO) Karaj Iran; ^2^ Department of Animal Science Payame Noor University Tehran Iran; ^3^ Department of Animal Science, Moghan College of Agriculture and Natural Resources University of Mohaghegh Ardabili Ardabil Iran; ^4^ Department of Animal Science, Faculty of Agriculture Ferdowsi University of Mashhad Mashhad Iran

**Keywords:** broilers, fat metabolism, Hypericum perforatum, immunity, malondialdehyde

## Abstract

**Background:**

Having bioactive components such as hypericin, hyperforin and quercetin has enabled Hypericum perforatum (HP) to show antioxidant, antiviral and hypocholesterolemic effects in different animal species.

**Objectives:**

This study aimed to investigate the effects of different levels of Hypericum perforatum extract (HPE) additions on performance, immune response, serum metabolites, and lipid peroxidation in broiler chickens.

**Methods:**

A total of 250 one‐day‐old broiler chicks were randomly allocated to five treatments with five replicates and ten chicks each. Experimental rations consisted of a basal diet with no supplement (control group) and a basal diet with 0.1%, 0.5%, 1%, and 1.5% HPE.

**Results:**

Results showed that the addition 1.5% HPE in the broiler diet increased high‐density lipoproteins (HDL) compared to the control diet. While the total cholesterol (TC) was significantly reduced by HPE supplementation, only feeding 1% and 1.5% HPE significantly lowered the low‐density lipoproteins (LDL). HPE addition to the diet significantly reduced abdominal and breast fat at 0.5%, 1%, and 1.5% levels. However, thigh fat was significantly decreased by dietary all HPE levels supplementation. Moreover, in the group fed with 1.5% HPE, the immunoglobulin G (IgG) and thymus weight significantly increased compared to the control group. Compared with the control group, HPE‐fed groups showed significantly lower malondialdehyde (MDA) concentrations in breast muscle. The total antioxidant capacity (T‐AOC) was improved significantly by HPE supplementation.

**Conclusions:**

In general, while there was no significant difference among treatments in the case of growth performance parameters, dietary supplementation of HPE at 1.5% was the most effective dose to improve serum biochemical metabolites, fat metabolism, immune response, and oxidative stability in broiler chicks.

## Abstract 2

Hypericum perforatum could be used as an antibiotic alternative in poultry nutrition due to its antioxidant, antiviral, and hypocholesterolemic effects. Results of this study showed that supplementation of HPE at 1.5% was the most effective dose to improve serum biochemical metabolites, fat metabolism, immune response, and oxidative stability in broilers.

## 1 Introduction

The presence of free radicals, including superoxide, hydrogen peroxide, and hydroxyl radicals, has been connected to the outbreak of many disorders in farm animals, such as sheep, cows, pigs, and poultry (Corino and Rossi 2021; Ahmadipour et al. 2024). Moreover, many studies have shown the adverse effects of reactive oxygen species (ROS) in poultry, such as DNA damage, protein oxidation, and lipid peroxidation in living tissues and cells resulting in poor immunity and retarded growth of birds (Righi et al. 2021).

Essential oils and phytogenic compounds such as flavonoids have been introduced as important antibiotic alternatives due to their lower toxicity, biodegradability and environmental compatibility (Liu et al. 2025). It has been demonstrated that biologically active components could promote the growth of poultry along with their antibacterial, antifungal, antiviral and emulsifying properties (Barreto et al. 2008; Mokhtari et al. 2023). These multi‐functional components are found in the whole parts of a plant, such as fruits, vegetables, nuts, grains, leaves, roots and barks; as reducing agents, they directly react with the quenching free oxygen radicals, or they can form chelating complexes with transition metals. Potentially, phenolic compounds can induce antioxidant enzymes or suppress the generation of oxidative enzymes such as cyclooxygenase in biological systems (Su et al. 2007). The most common plant phenolic antioxidants are flavonoids, cinnamic acid derivatives, coumarin, tocopherols, and multi‐functional organic acids (Moazzen et al. 2022).

Hypericum perforatum (HP) is also called St. John's wort and is traditionally used as a herbal tea and food supplement because of its excellent bioactive properties (Jarzębski et al. 2020). HP belongs to the family hypericaceae which is a perennial plant native to Europe, Asia, and North Africa. The flowers of this plant contain a red liquid composed of complex and biologically active compounds (Gupta et al., 2021; Jarzębski et al. 2020). It also appears that the translucent glands and surrounding tissues in leaves (from the upper to lower leaf epidermis) of HP contain the phytochemical hyperforin (Soelberg et al. 2007). HP contains at least ten classes of biologically detectable active compounds, mainly including a wide range of secondary metabolites such as naphthodianthrones (hypericin and pseudohypericin), phloroglucinols (hyperforin and adhyperforin), flavonoids (hyperoside, quercitrin, and isoquercitrin), tannins (tannic acid) and essential oils (terpenes and alcohols) (Jarzębski et al. 2020).

As a powerful antioxidant, St. John's wort can effectively inhibit the generation of free radicals and lipid peroxidation in different cells (Orčić et al. 2011). According to Zou et al. (2010), Hypericum perforatum extract (HPE) could decrease oxidative stress and prevent inflammation, neurotoxicity, and gastrointestinal problems. Landy et al. (2012) reported dietary addition of HP powder reduced TC in broilers. Moreover, HPE supplementation in broiler diets increased bird humoral immunity by increasing IgG (Aghili et al. 2014). Recently, Ilhan et al. (2024) have evaluated the effects of feeding powder and extract of HP on the growth and inflammatory responses of broiler chickens. They found that in comparison with the powder, the extract of HP significantly improved the body weight and immunity of broiler chickens.

The positive effects of HPE on the immunity and health of humans and rats are well documented. However, there are very few works regarding the application of this compound and its essential oil on the growth performance, immunity, and lipid peroxidation of broilers. Thus, this experiment aimed to investigate the effects of different levels of HPE on performance, serum metabolites, humoral immune response, and lipid peroxidation of broiler chicks.

## 2 Materials and Methods

### 2.1 Animal Husbandry and Dietary Treatment

A total of 250 one‐day‐old male broiler chickens (Ross 308) were randomly distributed into five dietary treatments with five replicates and ten birds each. Dietary treatments were a control diet containing maize‐soybean meal as basal diet and a basal diet supplemented with different levels of 0.1%, 0.5%, 1%, and 1.5% HPE. The starter, grower, and finisher diets were fed from 1 to 10 days, 11 to 24 days, and 25 to 42 days according to Aviagen recommendations for Ross 308 broiler chickens, respectively (Table 1). Diets were prepared for each period in the form of mash. Birds were reared in 25‐floor pens (1.0 × 1.0 m^2^) and had free access to feed and water throughout the trial. Each pen was covered with wood shavings at a depth of 5–8 cm, and equipped with a single tray feeder from 1–10 days, and suspended feeder from 11–42 days and a nipple drinker. The ambient temperature was maintained at 32°C for the first 3 days and then decreased by 2°C–3°C per week to reach 22°C, and after that was kept constant. The relative humidity was maintained at 50%–60%. The chicks were housed in an acclimatised room, with the provision of sufficient artificial light and ventilation. The lighting program was 23L: 1D for the first 7 days, 20L: 4D from 8 to 21 days, and 23L: 1D from 22 to 42 days. The ventilation rate was 0.12 m/s during the whole period.

**TABLE 1:** Ingredient and calculated composition of basal diets during starter, grower, and finisher periods.

Ingredients (g/kg)	Starter (0–10 days)	Grower (11–24 days)	Finisher (25–42 days)
Corn	50.19	53.43	59.28
Soybean meal	40.75	36.32	31.06
Soybean Oil	4.57	6.00	5.76
Dicalcium phosphate	1.75	1.72	1.60
Calcium carbonate	1.30	1.07	1.04
Common Salt	0.30	0.35	0.35
Vitamin‐ mineral premix^†^	0.50	0.50	0.50
DL‐Methionine	0.34	0.30	0.26
L‐Lysine	0.30	0.31	0.15
Total	100	100	100
Calculated composition			
ME (Kcal/Kg)	3025	3150	3200
Crude protein (%)	22.47	21.00	19.00
Calcium (%)	1.05	0.90	0.85
Available P (%)	0.50	0.45	0.42
Met + Cys (%)	1.07	0.95	0.86
Lysine (%)	1.43	1.34	1.09

*Note*: ^†^Premix provided per kilogram; Vitamin A: 5,500 IU; Vitamin D3: 1,500 IU; Vitamin E: 15 IU; Vitamin K: 8 mg; Thiamine: 1 mg; Riboflavin: 4 mg; Niacin: 25 mg; Biotin: 30 mg; Folic acid: 0.5 mg; Pantothenic acid: 5 mg; Pyridoxine: 1.5; Vitamin B12: 0.01 mg; Cu: 12 mg; Fe: 35 mg; Zn: 25 mg; Co: 0.15 mg; I: 0.5 mg; Se: 0.12 mg; Mn: 38 mg.

### 2.2 Preparation of Plant Extract

HP grows widely in the north, northwest, west and northeast regions of Iran, particularly in temperate and humid weather, with light sandy soil conditions and low humidity in plains and areas at low altitudes above sea level (Riazi et al. 2011). Following HP cultivation, its aerial parts were used for this study; these parts were collected during the flowering season (May to August), and 50 g of dried plant material was extracted using 1000 mL of water for 5 h (85°C, 800 rpm). The herb particles were then separated from the solution using a Whattman 10 µm filter paper. The extract was pre‐concentrated in a vacuum rotary evaporator at 60°C for 1 h. Then, the obtained extract was dried (24 h, 45°C) in a vacuum concentrator (Eppendorf, Germany). The residue was dissolved in distilled water and filtered using a 0.2 µm nylon filter, after which the filtrate was evaporated to dryness (Jarzębski et al. 2020).

### 2.3 Determination of Phenolic and Total Flavonoids Content of Hypericum Perforatum

The total content of phenols in plant extracts was determined by the Slinkard and Singleton method (Slinkard and Singleton 1977). Spectrophotometric methods were used to determine total phenolic and flavonoid contents, as already reported in earlier papers. Standard equivalents (gallic acid equivalent: GAE, for phenolics; rutin equivalent: RE, for flavonoids) were used to explain the contents in the plant extracts. Briefly, 0.5 mL of extract solution (0.01 g in 10 mL of methanol at 60°C) was mixed with 0.05 mL of 2% ammonium chloride. Then, 3 mL of 5% potassium acetate was added to the above solution. After 40 min, the absorbance of the samples was determined at 470 nm against distilled water. The absorbance of the samples was compared, and the total flavonoid content was expressed as 165.56 ± 2.4 mg per 100 g dry extract. The phenolic content in HPE was determined as 0.15%–0.30% hypericin, 1.4% hyperforin, and 0.40%–0.44% pseudohyperforin. Also, the content of total flavonoids, including quercetin, isoquercetin, rutin, hyperoside and quercitrine, was up to 3.50% (Table 2).

**TABLE 2:** Active substances of polyphenols found in the extract of Hypericum perforatum.

Extract group	Total phenolic compound (%)^†^	High yielding plant part
Naphthodianthrones	Hypericin (0.15–0.30)	Buds, flower parts and leaves
	Pseudohypericin (0.40–0.44)	
Phloroglucinols	Hyperforin (1.40)	Buds, flower parts, green fruit and translucent glands
	Adhyperforin (0.14–1)	
Flavonoids	Quercetin (1.50)	Buds, leaves and stems
	Hyperoside (0.55)	
	Quercitrin (0.52)	
	Isoquercitrin (0.40)	
	Rutin (0.42)	
Bioflavonoids	Biapigenin (0.09–0.45)	Flower parts
	Amentoflavone (0.01–0.04)	

*Note*:^†^Values are expressed as dry weight.

### 2.4 Growth Performance

The average daily weight gain (ADWG), average daily feed intake (ADFI), and feed conversion ratio (FCR) for each and the whole experimental period were recorded and used as measures of chick performance. The dead birds were weighed, recorded and used to adjust the FCR.

### 2.5 Serum Metabolites

At 42 days of age, two birds per replicate (10 birds/treatment) were randomly selected; blood samples were taken from a brachial vein, and were centrifuged at 3000 rpm for 15 min to obtain the serum (SIGMA 4–15 Lab Centrifuge, Germany). Each serum sample was decanted into Eppendorf tubes and stored at −20°C for later analysis. Serum triglyceride, TC, LDL, and HDL concentrations were measured spectrophotometrically using the Pars Azmoon kits (Pars Azmoon Inc., Tehran, Iran) and the enzymatic method.

### 2.6 Fat Deposition of Internal Organs

Fat deposition of internal organs (the liver, abdomen, and intramuscular tissue (thigh and breast)) was measured according to the methods described by Mirghelenj et al. (2016). For this, 2 g of breast, thigh, and liver were selected and dried in two 12 h stages (65°C, then 105°C) and cooled in a desiccator for at least 30 min. The liver, breast, and thigh intramuscular fat contents were measured by the Soxhlet system (AOAC, 1990) and expressed as percentages of dry tissue weight. Plus, the abdominal fat pad was removed and weighed as a percentage of live weight.

### 2.7 Antibody Response to Sheep Red Blood Cell

At 28 days of age, two birds in each group were randomly injected intramuscularly with 0.5 mL of 10% suspension of sheep red blood cells (SRBC) prepared in phosphate‐buffered saline. Blood samples were collected at 7 and 14 days after the first injection, and the total antibody titer against SRBC was evaluated using the Haemagglutination Inhibition Test. In total, mercaptoethanol‐sensitive (MES, presumably immunoglobulin M (IgM)) and mercaptoethanol‐resistant (MER, presumably IgG) anti‐SRBC antibody titres in serum samples were quantitated by the Cheema et al. (2003) method. All titres were expressed as log_2_ of the reciprocal of the highest dilution, giving visible Haemagglutination.

### 2.8 The Weights of Lymphoid Organs

At 42 days of age, two birds per replicate were selected randomly, slaughtered, plucked, eviscerated and then the thymus, spleen, and bursa of Fabricius were collected. Lymphoid organ weights were determined immediately after collection. Data were presented as a percentage of organ weight relative to total body weight.

### 2.9 Lipid Peroxidation

Breast tissue was sampled to determine the total antioxidant capacity (T‐AOC) activity and MDA concentration and stored at ‐20°C. The MDA concentration of serum and breast tissue, an index of lipid peroxidation and oxidative stress, was determined using the thiobarbituric acid reactive substances (TBARS) method. The absorbance was measured using the reaction of TBARS with MDA to create a stable pink colour with the spectrophotometer (UV‐2100, Unico Instruments Co., Shanghai, China) at 532 nm. Increased levels of MDA as a ROS indicate an effective indicator of lipid peroxidation in the body (Cortinas et al. 2005).

To determine T‐AOC, the frozen breast meat was homogenised, and the homogenate was centrifuged at 400 g for 10 min at 4°C. Then, the supernatants were stored at −70°C until analysis (Behroozlak et al. 2021). Finally, T‐AOC activities were measured by spectrophotometric using colorimetric methods and determined by spectroscopy (UNICO 7200, Shanghai, China) at a wavelength of 520 nm. These assays were performed using commercial assay kits produced by the Randox total antioxidant status kit (Randox Laboratories Ltd, Crumlin, UK) and their associated methods.

### 2.10 Statistical Analysis

Data were analysed in a completely randomised design using the general linear models (GLM) method of SAS. Significant differences among treatment means were separated using the Duncan multiple range test. The values of statistical significance were based on *P* < 0.05.

## 3 Results

### 3.1 Growth Performance

Table 3 shows the effect of different levels of HPE on FI, BWG, and FCR. During the starter, grower, finisher, and whole period, the addition of different dosages of HPE had no significant effect on FI, BWG, and FCR of broiler chickens (*P* > 0.05).

**TABLE 3:** Effects of different HPE^†^ levels on performance of broiler chickens.

	ADFI (g)^‡^				ADWG (g)^‡^				FCR (g/g)^‡^			
Treatments	0–10 days	11–24 days	25–42 days	0–42 days	0–10 days	11–24 days	25–42 days	0–42 days	0–10 days	11–24 days	25–42 days	0–42 days
Control	33.80	77.90	155.30	89.00	28.30	48.80	79.10	52.06	1.19	1.60	1.96	1.71
0.1% HPE	34.60	73.62	156.00	88.07	28.20	47.10	79.80	51.70	1.23	1.56	1.95	1.70
0.5% HPE	34.30	77.47	156.10	89.29	29.20	47.60	80.54	52.45	1.17	1.63	1.94	1.70
1% HPE	34.80	79.40	154.80	89.66	29.30	48.80	76.60	51.57	1.19	1.63	2.02	1.74
1.5% HPE	35.20	78.10	155.30	89.53	28.70	49.80	77.40	51.97	1.23	1.57	2.01	1.72
SEM^§^	0.80	3.16	0.83	3.56	0.86	3.16	6.17	3.18	0.11	0.07	0.06	0.14
*P*‐value	NS	NS	NS	NS	NS	NS	NS	NS	NS	NS	NS	NS

*Note*: ^†^HPE, Hypericum perforatum extract.

Abbreviations: ^‡^ADFI ‐ average daily feed intake; ADWG ‐ average daily weight gain; FCR ‐ feed conversion ratio.

^§^ SEM ‐ Standard Error of Means.

### 3.2 Serum Biochemical Metabolites

As shown in Table 4, all the HPE‐fed birds had lower TC and LDL than control birds (*P* < 0.05). However, the lowest LDL level was observed in 1% and 1.5% HPE‐fed birds (*P* < 0.05). Although the glucose and triglyceride concentrations of serum were not affected by dietary treatments (*P* > 0.05), consumption of 1.5% HPE resulted in higher HDL compared to the control diet (*P* < 0.05).

**TABLE 4:** Effects of different HPE^†^ levels on serum biochemical parameters of broiler chickens.

Treatments	Glucose (mg/dl)	TC^‡^ (mg/dl)	Triglyceride (mg/dl)	HDL (mg/dl)^‡^	LDL (mg/dl)^‡^
Control	242.00	133.50^a^	38.00	73.40^b^	31.52^a^
0.1% HPE	220.80	119.00^b^	35.80	75.20^b^	19.76^b^
0.5% HPE	216.40	114.60^b^	34.50	85.60^ab^	15.44^b^
1% HPE	212.40	102.00^b^	37.80	83.80^ab^	10.64^c^
1.5% HPE	214.80	101.80^b^	35.50	94.40^a^	7.60^c^
SEM^§^	9.59	6.45	8.79	4.83	1.55
*P*‐value	NS	0.04	NS	0.01	0.04

*Note*: ^a–c^Means within a column having different superscripts are significantly different (*P* < 0.05).

Abbreviations: ^†^HPE ‐ Hypericum perforatum extract.

^‡^ TC ‐ total cholesterol; HDL ‐ high density lipoprotein; LDL ‐ low density lipoprotein.

^§^ SEM ‐ Standard Error of Means.

### 3.3 Fat Deposition of Internal Organs

The effects of dietary addition of HPE on fat contents of the abdomen, liver, breast, and thigh muscles of chickens are presented in Table 5. No significant difference (*P* > 0.05) was observed in liver fat among treatments. Compared with the control group, feeding higher than 0.1% HPE significantly reduced abdominal and breast fat of broilers (*P* < 0.05). However, thigh fat was significantly decreased (*P* < 0.01) by dietary all HPE levels supplementation.

**TABLE 5:** Effects of different HPE^†^ levels on fat deposition of internal organs in broiler chickens.

Treatments	Abdominal fat (% of live weight)	Liver fat (g/kg DM)	Breast fat (g/kg DM)	Thigh fat (g/kg DM)
Control	2.21^a^	13.30	2.65^a^	5.83^a^
0.1% HPE	2.03^ab^	13.05	2.51^ab^	4.88^b^
0.5% HPE	1.85^bc^	12.22	2.49^b^	4.68^b^
1% HPE	1.90^bc^	12.89	2.44^b^	4.76^b^
1.5% HPE	1.65^c^	12.94	2.37^b^	4.55^b^
SEM^§^	0.14	0.28	0.05	0.08
*P*‐value	0.013	NS	<.001	<.001

*Note*: ^a–c^Means within a column having different superscripts are significantly different (*P* < 0.05)

Abbreviations: ^†^HPE ‐ Hypericum perforatum extract.

^§^ SEM ‐ Standard Error of Means.

### 3.4 Immune Response

Tables 6 and 7 show the relative weights of lymphoid organs and humoral immunity (SRBC test) of broilers, respectively. There was no significant difference (*P* > 0.05) in the weights of the spleen and bursa of Fabricius among treatments. However, the addition of 1.5% HPE to the diet significantly increased the thymus relative weight of birds (*P* < 0.05). No significant difference (*P* > 0.05) was observed in immunoglobulin T (IgT) and IgM in birds challenged with SRBC antigen at primary and secondary antibody titres, while only the addition of 1.5% HPE significantly (*P* < 0.01) increased IgG compared to the control group.

**TABLE 6:** Effects of different of HPE^†^ levels on lymphoid organs (% live body weight) of broiler chickens.

Treatments	BF^‡^	Thymus	Spleen
Control	0.13	0.22^b^	0.07
0.1% HPE	0.14	0.24^ab^	0.06
0.5% HPE	0.13	0.28^ab^	0.08
1% HPE	0.15	0.33^ab^	0.06
1.5% HPE	0.15	0.64^a^	0.07
SEM^§^	0.006	0.046	0.005
*P*‐value	NS	0.04	NS

*Note*: ^a–c^Means within a column having different superscripts are significantly different (*P* < 0.05).

Abbreviations: ^†^HPE ‐ Hypericum perforatum extract.

^‡^ BF ‐ Bursa of Fabricius.

^§^ SEM ‐ Standard error of means.

**TABLE 7:** Effects of different HPE^†^ levels on antibody response to SRBC (log_2_).

Treatments	Primary titre			Secondary titre		
	IgT	IgG	IgM	IgT	IgG	IgM
Control	4.70	1.20^b^	3.50	5.50	1.20^b^	4.10
0.1% HPE	4.70	1.20^b^	3.50	5.70	1.50^b^	4.20
0.5% HPE	5.30	1.50^b^	3.80	4.50	1.30^b^	3.20
1% HPE	5.30	1.70^b^	3.60	5.50	1.70^b^	3.80
1.5% HPE	5.90	2.50^a^	3.40	5.80	3.00^a^	2.80
SEM^‡^	0.40	0.24	0.37	0.48	0.23	0.37
*P*‐value	NS	0.007	NS	NS	0.0001	NS

*Note*: ^a–c^Means within a column having different superscripts are significantly different (*P* < 0.05).

Abbreviations: ^†^HPE ‐ Hypericum perforatum extract.

^‡^ SEM ‐ Standard error of means.

### 3.5 Lipid Peroxidation

Table 8 shows the effect of dietary treatments on MDA and T‐AOC values of serum and breast muscle of broilers at 42 days of age. Supplementing the basal diet with 1.5% and 1% HPE significantly (*P* < 0.001) increased serum or breast muscle T‐AOC activity. Serum MDA levels did not differ between experimental groups (*P* > 0.05). Compared to the control diet, dietary supplementation of HPE at any level significantly reduced the concentration of MDA in the breast muscle (*P* = 0.001).

**TABLE 8:** Effects of different HPE^†^ levels on lipid peroxidation of serum and breast muscle in broiler chickens.

Treatments	Serum		Breast muscle	
	MDA (µmol/L)	TAC (µmol/L)	MDA (µmol/mg protein)	TAC (µmol/g protein)
Control	6.35	6.34^c^	2.49^a^	2.37^c^
0.1% HPE	6.44	6.44^bc^	2.30^b^	2.41^bc^
0.5% HPE	6.39	6.54^b^	2.34^b^	2.40^bc^
1% HPE	6.41	6.77^a^	2.28^b^	2.45^ab^
1.5% HPE	6.44	6.80^a^	2.25^b^	2.49^a^
SEM^‡^	0.05	0.06	0.03	0.01
*P*‐value	NS	<.0001	0.001	<.0001

*Note*: ^a–c^Means within a column having different superscripts are significantly different (*P* < 0.05).

Abbreviations: ^†^HPE ‐ Hypericum perforatum extract.

^‡^ SEM ‐ standard error of means.

## 4 Discussion

### 4.1 Growth Performance

The experimental treatments had no effect on the growth performance parameters of broilers. In agreement with our results, Chikoto (2006) reported no significant effect of different levels of Ginkgo biloba herb and HPE on the performance of broilers. The effects of herbal supplementation on broilers depend on several factors, such as the derivatives, the part of the plant, the dose, the method, the number of additives (plant powder, essential oil, extract), and the rearing condition. For example, Ilhan et al. (2024) reported that chickens consumed feed with 1% powdered HP, and the 1.5 or 3 mL/kg of HPE level had no significant effect on growth performance compared to the chickens fed the control diet. Herbal additives and their by‐products can affect growth performance when birds are challenged by gut microbial loads such as an unclean environment, an unbalanced diet, and the presence of pathogenic microorganisms around the bird (Barreto et al. 2008), while the breeding environment of the present study was controlled and optimised. However, some studies indicated either negative or positive effects of HPE on the performance of broilers. In a study, Landy et al. (2012) stated that using 5 and 10 mg/kg HPE adversely affected the final body weight and feed conversion ratio of broilers. They suggested that some anti‐nutritional such as tannins found in HP, can bind proteins and reduce their absorption in digestive tracts, thus resulting in decreased growth performance. In contrast, in an experiment performed by Banisharif et al. (2016) and Davoodi et al. (2014), they observed that HPE supplementation improved the growth performance of broilers compared to the control group.

### 4.2 Serum Biochemical Metabolites

In accordance with our results, Hakimoğlu et al. (2007) observed that the use of the species of HPE called Lysimachioides lowered the TC and LDL due to the presence of phytosterols. Cholesterol is an essential structural element in biological membranes, and the rest is carried through the blood or functions as a building block for synthesizing bile acids, steroid hormones, and vitamin D (Zou et al. 2005). However, increased serum cholesterol concentration could increase the risk of developing cardiovascular diseases in broilers. The decrease in TC for HPE‐treated birds may be attributed to the inhibition of 3‐hydroxy‐3‐methylglutaryl coenzyme A (HMG CoA) activity or increased excretion of bile acids and faecal cholesterol (Zou et al. 2005; Asgary et al. 2012). Flavonoids, glycosides, and sterols found in HPE have shown hypocholesterolemic effects and improved the efficiency of fat metabolism (Raso et al. 2002, Zou et al. 2005). Feeding more than 0.5% HPE led to a decrease in serum LDL value, which may be related to higher phenolic compounds in HPE. Similar to our results, Davoodi et al. (2014) showed that LDL levels were significantly reduced in the HPE‐fed groups while HDL levels were increased. In rats, the extract of HP reduced the serum lipid profile, including LDL cholesterol and total cholesterol (Ghosian Moghaddam et al. (2016). It has been speculated that HP is a natural bile laxative that increases the bile flow (choleretic effect) and excretes excess bile by faeces (cholagogue effect), subsequently inhibiting the increase in blood serum cholesterol (Mohagheghzadeh et al. 2023).

### 4.3 Fat Deposition of Internal Organs

While HPE supplementation at all levels significantly reduced thigh fat, feeding HPE higher than 0.1% significantly reduced the abdominal and breast fat of broilers. In agreement with this study, Davoodi et al. (2014) and Hosseini et al. (2015) reported that drinking water supplementation of HPE to broiler chicks at graded levels reduced abdominal fat percentage compared with the control group. Contrary to our results, the study by Hristakieva et al. (2021) demonstrated that the inclusion of a 1% dry form of HP in the diet had no significant effect on the fat content of abdominal fat, breast, and thigh tissues of turkey. Based on the previous findings (Hsu et al. 2009; Tian et al. 2015; Mohagheghzadeh et al. 2023), it seems that instead of the herbal form, using the extract form of HP can reduce fat deposits in animal tissues due to the presence of flavonoids, bioflavonoids, phenylpropanoids, phloroglucinols, naphthodianthrones and perhaps its secondary metabolites such as hypericin, pseudohypericin, hyperforin, adhyperforin, and rutin. Another possible explanation could be related to CPT‐1 (an important regulator of fatty acid oxidation) activation and inactivation of FATP‐1 (major transporter of fatty acid into cells) expression, which reduces lipid accumulation in skeletal muscle as it was reported in mice previously (Tian et al. (2015).

### 4.4 Immune Response

There was no significant difference in the weights of the spleen and bursa of Fabricius among treatments, which is in agreement with the results of Hristakieva et al. (2023). However, adding 1.5% HPE to the diet significantly increased the thymus relative weight of birds. In accordance with our results, Shang et al. (2012) reported a lower spleen index for the challenged IBDV (infectious bursal disease virus) chickens as compared to the high‐dose HPE‐treated group, in which chicks challenged with IBDV were administrated with hypericin as the main active ingredient in HPE. It has been proposed that the bioactive substances (hypericin and hyperforin) found in HPE may have potent antiviral and anti‐inflammatory effects in broiler chicks (Ilhan et al. 2024). IgG significantly was increased by the addition of 1.5% HPE to the diet. Similar results were reported for rats that received increased levels of HPE in diets Aghili et al. (2014). The possible explanation for this enhancement could be related to the significant proliferation of immune cells in the organs of the immune system (Aghili et al. 2014). Also, Landy et al. (2012) showed that adding 5 and 10 g/kg of HP from dried aerial parts to the diet significantly increased the antibody titres against the influenza virus in serum in broiler chickens. The polyphenolic and bioactive ingredients in HP, such as hyperforins, flavonoids, and hypericins in the extract can enhance the humoral immunity of birds by increasing IgG levels in serum (Shang et al. 2012).

### 4.5 Lipid Peroxidation

Dietary supplementation of HPE from low to high dosage significantly reduced the concentration of MDA only in the breast meat. Raw chicken meat with high levels of unsaturated fatty acids can be sensitive to lipid peroxidation, especially when broilers are fed vegetable oils during storage (Abbasi et al. 2019). On the other hand, free radicals such as superoxide anion (O_2_), hydrogen peroxide (H_2_O_2_), and hydroxyl radical (OH.), which cause lipid peroxidation, can lead to cell death or apoptosis in many cells (Martemucci et al. 2022). Lipid peroxidation occurs during the processing and storage of meat and meat products, which adversely affects the colour, taste, texture, and nutritional value of meat, leading to lower consumer interest in purchasing meat products (Behroozlak et al. 2021). Decreased serum MDA levels could be associated with increased free radical scavenging capacity and reduced tissue or cell damage with HPE supplementation. Due to the wide variety of active components in herbs and plant extracts, these compounds can extend the shelf life and improve the quality of meat products (Kürekci et al. 2021). Recent findings have shown that several antioxidant compounds, such as polyphenols, flavonoids, and terpenes, could prevent or reduce ROS‐induced damage (Tungmunnithum et al. 2018). Dong et al. (2020) showed that dietary quercetin (from 200 to 800 ppm) alleviated the increase of the MDA level in serum and improved the activity of GPX (at 800 ppm) on day 11. Quercetin (formed 1.5% of our extract samples) is considered one of the secondary metabolites of HPE that could alleviate H_2_O_2_‐induced cell damage, reduce the intracellular ROS level, and reverse the change of MDA levels (Chen et al. 2018). The overall antioxidant effect of alcoholic HPE on lipid peroxidation is related to scavenging hydroxyl and chelation of divalent metal ions (Benedi et al. 2004), which inhibits the superoxide and other ROS production (Silva et al. 2008). It has been reported that hypericin, as the main compound of HPE, induces ubiquitinylation of the Hsp90 (the heat shock protein 90) chaperone, playing an important role in protecting the cells against oxidative stress (Behboodi et al. 2021). Adding 1.5% and 1% HPE to the diet significantly increased serum or breast muscle T‐AOC activity. Not only do herbal supplements show antioxidant activity, but they also improve the oxidative stability of animal products (Bhatt 2015). Similarly, El‐Sherbiny et al. (2003) found that the inclusion of HPE (containing flavonoids with documented antioxidant activity) in mice suppressed lipid peroxidation by reducing MDA concentration along with increasing the activity of glutathione peroxidase (GPX) and glutathione (GSH) in brain cells. In the present study, an explanation for the increase in breast and serum antioxidant capacity in broilers fed HPE is that flavonoid compounds such as quercetin elevate the serum ferric reducing antioxidant power (FRAP) and the content of small antioxidant molecules such as ascorbic acid and vitamin E in body cells (Zhao et al., 2011). As far as we examined, very few studies have been published on the effect of HPE supplementation on lipid peroxidation in broiler chicks. Therefore, further experiments are needed to investigate the effects of HPE on the lipid peroxidation and antioxidant capacity in broiler chicks.

## 5 Conclusions

This study showed that while the growth performance of birds was not adversely affected by HPE supplementation, feeding HPE reduced the TC status and thigh, breast, and abdominal fat of broiler chicks. Moreover, supplementing the basal diet with 1.5%, HPE caused an improvement in the humoral immune response. The effect of HPE supplementation on lipid peroxidation status was more pronounced in meat rather than blood serum, showing lower MDA at all HPE doses and higher TAC at 1% and 1.5% HPE levels. According to the results, the dietary addition of HPE at 1.5% was the most effective level to improve serum biochemical metabolites, fat metabolism, immune response, and oxidative stability in broiler chicks.

## Author Contributions

**Mohammad Ali Behroozilak**: investigation, writing–original draft, methodology, writing–review and editing, software, formal analysis, conceptualization, supervision, data curation. **Seyed Adel Moftakharzadeh**: investigation, writing–original draft, methodology, writing–review and editing, software, formal analysis, project administration, supervision. **Morteza Behroozlak**: conceptualization, validation, funding acquisition, data curation, resources, formal analysis, investigation. **Vahid Vahedi**: funding acquisition, methodology, formal analysis, resources, data curation, visualization. **Somayeh Bakhshalizadeh**: Writing–review and editing, formal analysis, software, data curation, resources, methodology.

## Ethics Statement

All the experimental proceedings in this experiment were approved by the university animal ethics committee.

## Conflicts of Interest

The authors declare no conflicts of interest.

## Peer Review

The peer review history for this article is available at https://publons.com/publon/10.1002/vms3.70507

## Data Availability

Data supporting the findings of this study are available upon request from readers via the email address provided.
